# Occupational accidents with exposure to biological material in a university hospital in Pernambuco

**DOI:** 10.47626/1679-4435-2023-1106

**Published:** 2024-11-14

**Authors:** Edivane Patrícia Galdino Monteiro, Ivanéle Maria Soares Bezerra, Magaly Bushatsky, Adriana Conrado de Almeida, Rosana Anita da Silva Fonseca, Emanuel Sávio de Souza Andrade

**Affiliations:** 1 Master’s in Forensic Expertise, Universidade de Pernambuco (UPE), Recife, PE, Brazil; 2 Nossa Senhora das Graças School of Nursing, UPE, Recife, PE, Brazil; 3 Coordination of the Master’s in Forensic Expertise, School of Dentistry, UPE, Recife, PE, Brazil; 4 Institute of Biological Sciences, UPE, Recife, PE, Brazil; 5 Master’s in Forensic Expertise, School of Dentistry, UPE, Recife, PE, Brazil

**Keywords:** accidents, occupational, occupational exposure, occupational health, acidentes de trabalho, exposição ocupacional, saúde do trabalhador

## Abstract

**Introduction:**

Occupational accident with biological material consists of the worker’s contact with blood and/or other organic fluids during their working shift.

**Objectives:**

To describe the epidemiological profile of occupational accidents with exposure to biological material reported in a university hospital in Pernambuco, Brazil, from 2009 to 2019.

**Methods:**

This is an analytical, cross-sectional, retrospective study that analyzes 134 notification forms of accidents with exposure to biological material. Bivariate analysis was performed with Fisher’s exact test.

**Results:**

Women had more accidents, 83.6% (n = 112). Nursing professionals represented 45.5% (n = 61) of cases. Percutaneous exposure predominated in 70.9% (n = 95). Drug administration and inadequate disposal of sharps accounted for 62.7% (n = 84), and gloves were the most worn personal protective equipment, 62.7% (n = 84). Of those injured, 62.7% (n = 84) had been fully vaccinated against hepatitis B.

**Conclusions:**

This study refers us to the relevance of broad and comprehensive information about worker health policies, including technical-professional foundation, labor rights, the importance of records, and prevention awareness.

## Abstract (pt)

**Introdução:**

O acidente de trabalho com material biológico consiste no contato do trabalhador com sangue e/ou outros fluidos orgânicos durante a jornada laboral.

**Objetivos:**

Descrever o perfil epidemiológico dos acidentes de trabalho com exposição a material biológico notificados em um hospital universitário do estado de Pernambuco no período de 2009 a 2019.

**Métodos:**

Foi realizado um estudo analítico, transversal, retrospectivo, a partir da análise das 134 fichas de notificação dos casos de acidentes com exposição a material biológico. Foi realizada análise bivariada aplicando o teste exato de Fisher.

**Resultados:**

Ficou evidenciado o predomínio de acidentes envolvendo mulheres, com 83,6% (n = 112) dos casos. Os profissionais da enfermagem corresponderam a 45,5% (n = 61) dos casos. A exposição percutânea foi a mais predominante, correspondendo a 70,9% (n = 95) dos casos. Procedimentos para administração de medicações e o descarte inadequado de material perfurocortante foram responsáveis por 62,7% (n = 84) das ocorrências. Entre os equipamentos de proteção individual, o mais utilizado foi a luva, com 62,7% (n = 84), e o esquema vacinal contra a hepatite B estava completo em 62,7% (n = 84) dos acidentados.

**Conclusões:**

O estudo nos remete à relevância da informação ampla e abrangente para as políticas da saúde do trabalhador, incluindo o alicerce técnico-profissional, direitos trabalhistas, a importância dos registros e conhecimentos quanto à prevenção.

## INTRODUCTION

Occupational accident is defined as events that occur while working for a company or a domestic employer and result in injury or functional impairment, death, or loss/reduction, permanent or temporary, of work ability.^1^ Accidents at work can be: commuter accidents, when they occur on the route between home and work, or vice versa; occupational diseases or common accidents, when produced or triggered while working; and occupational diseases, produced or triggered by special conditions when working, directly relating to it.^1-3^

According to the Ministério do Trabalho e Emprego (Brazil Ministry of Labor and Employment) e o Ministério da Previdência Social (Brazil Ministry of Social Welfare),^3,4^ 375,300 (63.95%) of the 586,857 occupational accidents reported in Brazil in 2019 were common accidents, 99,118 had no *comunicação de acidente de trabalho* (CAT, occupational accident report form), and about 5.15 of every 100,000 were fatal accidents. In 2019, 14,396 accidents were reported, 7,193 were common, and 4,432 had no CAT in Pernambuco, Brazil.^3^

According to the Ministério da Saúde (Brazil Ministry of Health),^5^ occupational accidents involving biological material are defined as direct or indirect exposure, on the working shift, to organic material potentially contaminated with pathogens.

In Brazil, concerns with prophylactic measures and clinical and laboratory monitoring for health care personnel exposed to the risk of occupational accidents only began to increase after the HIV/AIDS infection epidemic in the early 1980s, but still very incipiently.^5^

In cases of exposure to biological material, the Ministério da Saúde^6,7^ has gradually developed protocols for professionals exposed to risk, such as the Protocolo Clínico e Diretrizes Terapêuticas para Profilaxia Pós-Exposição de Risco à Infecção pelo HIV, Infecções Sexualmente Transmissíveis e Hepatites Virais (Clinical Protocol and Therapeutic Guidelines for Post-Exposure Prophylaxis against HIV, Sexually Transmitted Infections, and Viral Hepatitis) and the Protocolo de Exposição a Materiais Biológicos (Protocol for Exposure to Biological Material). In addition, it aims to determine that health services should define written protocols with clear procedures for occupational accidents.^2,8^

A medical examination is essential in the processes of *licença médica para tratamento de saúde* (LMTS, sick leave for health treatment), disability retirement, readaptation, accident nexus, occupational disease, and work-related disease, among other legal provisions. This is also fundamental in the decision-making process as to the length of time away from work in the event of an injury.^9^

Considering the significant lack of reporting and notification of these accidents in Brazil, some studies indicate that approximately 50% of these exposures are underreported.^2,10^ A study with a sample of 747 accidents showed that 71% were not reported.^11^ The rate of clinical and laboratory follow-up dropout is also significant.^12^

This study aims to describe the epidemiological profile of accidents with exposure to biological material reported at a university hospital in Pernambuco, Brazil, between 2009 and 2019.

## METHODS

The Research Ethics Committee approved this study (Certificate of Submission for Ethical Appraisal: No. 62842722.3.0000.5207), following all the ethical precepts set out in Resolução do Conselho Nacional de Saúde/Ministério da Saúde No. 466/2012 (Brazil National Health Council/Ministry of Health Resolution No. 466/2012), Norma Operacional 001/2013 (Operational Standard 001/2013), and the principles of the Lei de Ambiente Virtual (Virtual Environment Law) (Carta Circular No. 01/2021). The informed consent letter was obtained and the Informed Consent Form was waived.

This is an analytical, retrospective, cross-sectional epidemiological study. The study population consisted of all records of occupational accidents involving exposure to biological material between 2009 and 2019 at a university hospital in the state of Pernambuco, Brazil. The sample is based on reported incidents, totaling 134 notification forms. Therefore, no records were excluded and all those identified during the study were recorded.

Data were collected using a form, based on the notification form for accidents involving exposure to biological material (a template provided by the Sistema Nacional de Agravos de Notificação [SINAN, Brazil Notifiable Diseases Information System]). The data were double entered to minimize risks and ensure the completeness of the information collected, and then stored in an online spreadsheet. Initially, the relative and absolute frequencies of the categorical variables and central measure, position, and dispersion statistics for the numerical variables were computed.

Bivariate analysis was performed to see if a statistical relationship existed between some of the variables (all categorical). The Fisher’s exact test was used, with statistical significance considered for p < 0.05.

A limitation of the study included numerous variables with a high amount of missing data, and many of them was not included in the data analysis.

The categorization of data completeness by Abath et al.^13^ was used to identify which variables would be included in the investigation: good - greater than 75%; regular - greater than 50% and less than or equal to 75%; low - greater than 25% and less than or equal to 50%; very low - less than or equal to 25%. Variables with good categorization (up to 25% missing data) were considered.

R 4.1.0 was used for all calculations. The level of significance for the study was set at 5%.

## RESULTS

The study used a sample of 134 notification forms for occupational accidents involving exposure to biological material, which were notified and followed up at the institution (Table 1).

**Table 1:** Descriptive statistics of the variables (n = 134)

Variable/categories	n	%
Sex		
Male	22	16.4
Female	112	83.6
Ethnicity/skin color^*^		
Brown/Yellow/Indigenous	70	52.2
White	27	20.1
Black	12	9.0
Occupation†		
Laboratory workers	16	11.9
Nursing professionals	61	45.5
Students/residents	20	14.9
Cleaners	20	14.9
Other	11	8.2
Employment situation‡		
Permanent employee	24	17.9
Unemployed/temporary worker	9	6.7
Civil servant	41	30.6
Other	29	21.6
Type of exposure (percutaneous)§		
No	19	14.2
Yes	95	70.9
Circumstance of the accident^||^		
Inadequate disposal of sharps	32	23.9
Other	7	5.2
Surgical/laboratory procedure	10	7.5
Drug administration	52	38.8
Agent¶		
Needles with a lumen	73	54.5
Needles without a lumen	6	4.5
Blade/lancet (any type)	14	10.4
Other	27	20.1
PPE (gloves)^**^		
No	29	21.6
Yes	84	62.7
PPE (apron)††		
No	78	58.2
Yes	30	22.4
PPE (goggles)‡‡		
No	92	68.7
Yes	12	9.0
PPE (mask)§§		
No	62	46.3
Yes	44	32.8
Vaccination status (hepatitis B)^||||^		
Not vaccinated	20	14.9
Vaccinated	84	62.7
Age		
Low	19	
Median	36	
Mean	37	
High	70	
Standard deviation	11.3	
Coefficient of variation	30.2%	

PPE = personal protective equipment.

Missing data:

* 18.7% (n = 25); †4.5% (n = 6); ‡23.1% (n = 31); §14.9% (n = 20); ||24.6% (n = 33); ¶10.4% (n = 14);

** 15.7% (n = 21); ††19.4% (n = 26); ‡‡22.4% (n = 30).30); §§20.9% (n = 28); ||22.4% (n = 30).

The results showed that 83.6% (n = 112) of the injured were women and 16.4% were men. As for ethnicity/skin color, 52.2% (n = 70) were Brown, Yellow or Indigenous (the latter two were represented by only one individual each), 21% were White, and 9% were Black. The mean age was 37, ± 11 years, from 19 to 70 years old.

In terms of epidemiological background, 45.5% (n = 61) were nursing professionals. Among students/residents and cleaners, 14.9% (n = 20) of the accidents were recorded in each category. Laboratory workers were 11.9% (n = 16) of the sample.

Also, 30.6% (n = 41) of the injured were civil servants, 21.6% (n = 29) were classified as other, not described on the notification form. In addition, 17.9% (n = 24) were permanent employees, while 6.7% (n = 9) were temporary workers or unemployed.

The category accident with biological material recorded most of the information directly related to the reported event, with the following results: 70.9% (n = 95) had percutaneous exposure, and in 14.2% (n = 19) of the cases the biological material came into contact with mucous membranes, intact skin, or non-intact skin. Also, exposure to blood was recorded in 73 cases, although this issue was not fully analyzed because 26.1% of the records lacked this variable. Drug administration corresponded to 38.8% (n = 52) of the accident circumstances.

The agent of 54.5% (n = 73) of the cases was needles with a lumen; blades and lancets caused 10.4% (n = 14) of the incidents; 4.5% (n = 6) were with needles without a lumen; other agents caused 20.1% (n = 27) of the notifications, and the agent was not reported on the notification form. As for the use of personal protective equipment (PPE), 62.7% (n = 84) wore gloves; 22.4% (n = 30) wore aprons; 9% (n = 12) wore goggles; and 32.8% (n = 44) wore masks. Furthermore, 62.7% (n = 84) of the injured patients were vaccinated against hepatitis B.

Other variables, such as education background, length of service, examination of the injured patient, known source patient, examination of the source patient, approach at the time of the accident, history of the case, and notification with a CAT were not considered for statistical analysis due to the high percentage of missing data on the notification forms.

When comparing the variables involving the accident circumstances, agent, PPE (gloves), type of exposure (percutaneous), and occupation (Table 2), the following results were significant: when the accident circumstances were improper disposal of the material, cleaners are more likely to be involved (94.4%), while other workers accounted for between 13.3% and 20%. When the accident circumstances were a surgical/laboratory procedure, most of those injured were laboratory workers (30.8%). The highest rate found in other occupations was 20%, but this refers to a single individual.

**Table 2:** Bivariate analysis of the variables accident circumstances, agent, PPE (gloves), type of exposure (percutaneous), and occupation

Variable/categories	Occupation										p-value
	Laboratory workers		Nursing professional		Students/residents		Cleaners		Other		
	n = 16	%	n = 61	%	n = 20	%	n = 20	%	n = 11	%	
Accident circumstance											
Inadequate disposal of sharps	2	15.4	9	20.0	2	13.3	17	94.4	1	20.0	0.00
Other	0	0.0	4	8.9	1	6.7	1	5.6	1	20.0	
Surgical/laboratory procedure	4	30.8	3	6.7	2	13.3	0	0.0	1	20.0	
Drug administration	7	53.8	29	64.4	10	66.7	0	0.0	2	40.0	
Agent											
Needle with lumen (light)	10	76.9	34	61.8	13	65.0	13	72.2	1	11.1	0.00
Needle without lumen	0	0.0	0	0.0	2	10.0	1	5.6	2	22.2	
Blade/lancet (any type)	0	0.0	5	9.1	3	15.0	3	16.7	3	33.3	
Other	3	23.1	16	29.1	2	10.0	1	5.6	3	33.3	
PPE (gloves)											
No	4	33.3	17	30.9	1	5.9	4	25.0	3	37.5	0.22
Yes	8	66.7	38	69.1	16	94.1	12	75.0	5	62.5	
Type of exposure (percutaneous)											
No	1	6.7	12	23.5	1	5.6	1	5.3	2	33.3	0.11
Yes	14	93.3	39	76.5	17	94.4	18	94.7	4	66.7	

PPE = personal protective equipment.

In drug administration, nearly 65% of accidents occurred among nursing professionals or students/residents. This proportion was 20% to 62.5% higher when compared to the other occupations (p < 0.001). The agent needles with a lumen injured around 20% more laboratory workers and cleaners than nursing professionals or residents/students (p < 0.001). The other agents were not sufficiently significant to correlate (p > 0.05).

A comparison of the accident circumstances with the type of agent (Table 3) found that improper disposal of sharps is more frequent when the type of agent is a blade/lancet (any type) and/or needles without a lumen, which is twofold or more compared to the other agents. In drug administration, the most common agent is needles with a lumen, which is 90% more frequent than needles without a lumen, and 35% more frequent than other agents; the blade/lancet (any type) has no frequency at all.

**Table 3:** Comparison between the accident circumstances and the agent type

Variable/categories	Agent								p-value
	Needles with a lumen (light)		Needles without a lumen		Blade/lancet (any type)		Other		
	n = 73	%	n = 6	%	n = 14	%	n = 27	%	
Accident circumstance									
Inadequate disposal of sharps	20	29.4	3	50.0	6	66.7	2	13.3	0.00
Other	2	2.9	1	16.7	0	0.0	4	26.7	
Surgical/laboratory procedure	3	4.4	0	0.0	3	33.3	2	13.3	
Drug administration	43	63.2	2	33.3	0	0.0	7	46.7	

Table 4 shows the correlation between the accident circumstances and the type of exposure (percutaneous), and whether the professional wore PPE (gloves). No significant relationship was found.

**Table 4:** Correlation between the accident circumstances and the type of exposure (percutaneous), and whether the professional wore PPE (gloves)

Variable/categories	PPE (gloves)				p-value
	No		Yes		
	n = 29	%	n = 84	%	
Accident circumstance					
Improper disposal of sharps	8	36.4	19	26.8	0.31
Other	2	9.1	5	7.0	
Surgical/laboratory procedure	0	0.0	9	12.7	
Drug administration	12	54.5	38	53.5	
Type of exposure (percutaneous)					
No	6	22.2	13	17.6	0.58
Yes	21	77.8	61	82.4	

PPE = personal protective equipment.

## DISCUSSION

This study found that women were more likely (83.6%; n = 112) to report accidents involving biological material. Other studies^14,15^ have also shown similar results, with 93.4% and 79.43% of accidents involving women, respectively.

This study found that nursing professionals reported 45.5% (n = 61) of the notifications. The Fisher’s exact test for the occupation *versus* accident circumstances showed that nursing professionals were significantly more likely to be exposed to accidents with biological material. Another study, in which the nursing professionals represented 25%, found that 76% reported having had an occupational accident with sharps.^11^ Nursing professionals have also been referred to in other studies as a highly vulnerable category due to their duties, as they provide direct and permanent assistance to patients.^16,17^

Drug administration accounted for 38.8% (n = 52) of the accidents. These include drug administration by various routes, fingerstick glucose test, and needle re-tapping, which are primarily performed by nursing professionals, and blood collection for laboratory or non-laboratory purposes, which can be performed by other professionals. In a study in a reference hospital in Tocantins, Brazil, 22% of accidents involving exposure to biological material were caused during this procedures.^18^

In this study, the percutaneous route was the most prevalent type of exposure, representing 70.9% of cases. This rate is consistent with the literature, which also points to the percutaneous route as the most common, with studies showing similar rates of 74.15% and 76.8% of cases, respectively.

The variable accidents due to improper disposal of sharps is particularly important, with a rate of 23.9% (n = 32). This figure is almost threefold higher than the total reported in the study by Bordin et al.^15^ (8.51%) and almost twofold higher than in the study by Gonçalves et al.^19^ (13.51%).

In this study, a statistically significant relationship was found between occupation and agent. The rate of accidents caused by needles with a lumen was 76.9% (n = 10) in laboratory workers and cleaners, around 20% higher than in nursing professionals and students. Needles with a lumen were reported as a major causative agent, at 64.5% of accidents with exposure to biological material in a study in Rio Grande do Sul, Brazil.^14^

In this study, 62.7% (n = 84) of the injured workers wore mainly gloves and were vaccinated against hepatitis B. In the aforementioned study, gloves were also the most commonly worn PPE (75.2%), and workers were vaccinated in 93.4% of cases.^14^ The Agência Nacional de Vigilância Sanitária (ANVISA, Brazil National Health Surveillance Agency) refers to precautionary measures, recommending hand hygiene as a primary measure for infection control, proper waste disposal as a safety measure, and wearing PPE according to the risk of exposure.^20,21^

In this study, blood was present in 54.5% (n = 73) of accidents; however, it was not possible to assess this variable statistically, given the large number of cases without this information on the notification form. According to a study at a university hospital in the state of Rio de Janeiro, Brazil, blood was the most common organic material in accidents (94.06%).^19^

Missing or incomplete data was considered a limiting factor in this study, thus making it difficult to analyze the variables intended to compose the epidemiological profile. Incomplete records and underreporting are factors that interfere with the statistical analysis of the data, and are limitations of the study.^15^

In this study, we failed to identify the progress of the cases, as 95% of them missed information. Bordin et al.^15^ found that follow-up dropout occurred in 23.40% of cases. Sardeiro et al.^12^ reported that follow-up dropout was 41.5%, directly related to nursing professionals, dentists, or cleaners over the age of 40. In these cases, the agent was not needles with a lumen, the professionals were permanent employees, had no CAT, and refused HIV post-exposure prophylaxis.^12^

In this study, 97.7% (n = 132) of the respondents failed to fill in the information about their CAT. According to the Ministério do Trabalho e Emprego^22^ and Decree No. 3.048,^23^ this is a mandatory document for companies, which are required to inform the Previdência Social (Brazil Social Security) of occupational accidents, commuting accidents, or occupational diseases, and immediate notification is mandatory in the event of a work-related death. In Brazil, approximately 17% of accidents with exposure to biological material have a CAT. In 2013, it was estimated that only one out of seven accidents was reported.^3,4^

This study reveals the importance of recommendations for the prevention and control of infectious and parasitic diseases. The incentive to vaccinate against preventable diseases/infections stands out, especially health care personnel. In addition, it is essential to underline the importance of educational practices that demonstrate the value of the quality of the information reported and CAT, not to mention actions aimed at preventing accidents, including wearing PPE and proper waste disposal.

It was clear that it is essential for workers to have access to accurate information and to the rights assigned to the job. It is essential to be aware of individual and collective actions to prevent illnesses, risks associated with occupational practice, including biological risks, and to ensure a healthy workplace.^24^

This consolidates the relevance of practices and the institutionalization of policies aimed at occupational health. Missing data were found as a limitation in a number of studies on the subject, similarly to what we found in this one. Directive work with the team assigned to provide care, which includes guidance and justifying the importance of the information contained in the notification forms drawn up by health management bodies, is crucial for the formulation of public policies adapted to real circumstances.

## CONCLUSIONS

Most accidents with exposure to biological material was with nursing professionals, women, with brown skin, and a mean age of 37 years.

Percutaneous injury was the most common type of exposure, particularly needle sticks. Procedures involving drug administration and improper disposal of sharps were significant in accidents with exposure to biological material. Gloves were found to be the most commonly worn PPE, and most of the injured patients were vaccinated against hepatitis B.

The bivariate analyses showed a significant correlation between nursing-related activities, such as drug administration and accidents with biological material. Similarly, accidents with needles with a lumen were associated with laboratory workers and cleaners, especially improper disposal of sharps in the latter.

Missing data was considered a limiting factor in this study, making it impossible to statistically analyze some variables, including case progress and CATs.

This study reminds us of the importance of broad and comprehensive information for workers’ health policies, serving as a technical foundation for the injured professional and the person responsible for their care. This approach includes the need for updates on clinical approaches and necessary follow-up, labor rights, expert opinions, and benefits. Additionally, it stresses the importance of records, such as the notification form and the CAT, and prevention awareness, including vaccination, proper use of PPE, and promoting safe practices.
